# Simply Imagining Sunshine, Lollipops and Rainbows Will Not Budge the Bias: The Role of Ambiguity in Interpretive Bias Modification

**DOI:** 10.1007/s10608-013-9564-x

**Published:** 2013-07-24

**Authors:** Patrick J. F. Clarke, Shenooka Nanthakumar, Lies Notebaert, Emily A. Holmes, Simon E. Blackwell, Colin MacLeod

**Affiliations:** 1 School of Psychology, University of Western Australia, Crawley, WA 6009 Australia; 2 MRC Cognition and Brain Sciences Unit, Cambridge, UK; 3 School of Psychology, Babes-Bolyai University, Cluj-Napoca, Romania

**Keywords:** Interpretive bias, Interpretive bias modification, CBM-I, Imagery, Ambiguity

## Abstract

Imagery-based interpretive bias modification (CBM-I) involves repeatedly imagining scenarios that are initially ambiguous before being resolved as either positive or negative in the last word/s. While the presence of such ambiguity is assumed to be important to achieve change in selective interpretation, it is also possible that the act of repeatedly imagining positive or negative events could produce such change in the absence of ambiguity. The present study sought to examine whether the ambiguity in imagery-based CBM-I is necessary to elicit change in interpretive bias, or, if the emotional content of the imagined scenarios is sufficient to produce such change. An imagery-based CBM-I task was delivered to participants in one of four conditions, where the valence of imagined scenarios were either positive or negative, and the ambiguity of the scenario was either present (until the last word/s) or the ambiguity was absent (emotional valence was evident from the start). Results indicate that only those who received scenarios in which the ambiguity was present acquired an interpretive bias consistent with the emotional valence of the scenarios, suggesting that the act of imagining positive or negative events will only influence patterns of interpretation when the emotional ambiguity is a consistent feature.

## Introduction

The tendency to resolve ambiguous information in favour of negative meanings is consistently implicated in the aetiology and maintenance of anxiety and depression by cognitive models of emotional pathology (e.g. Clark and Beck 2010; Williams et al. 1997). Research seeking to directly alter patterns of selective interpretation supports the presence of a causal relationship between this cognitive bias and emotional vulnerability. Such research has consistently demonstrated that the reduction of interpretive bias favouring negative resolutions of ambiguity also reduces emotional vulnerability (cf. MacLeod and Mathews 2012). These promising experimental findings have highlighted the possibility that cognitive bias modification for interpretation (CBM-I) could potentially deliver applied benefits in real-world settings.

Imagery-based CBM-I represents a relatively new approach to traditional CBM-I techniques and is being increasingly adopted as a means of altering selective interpretation. Unlike traditional CBM-I approaches that require the active resolution of ambiguous scenario content (e.g. fragment completion), imagery-based CBM-I commonly involves auditory presentation of scenarios depicting everyday events, requiring listeners to actively imagine the situations described using a first person perspective (Holmes and Mathews 2005). The scenarios are constructed such that they are initially emotionally ambiguous, with the emotional tone of the situation only becoming apparent in the final word/s. An example of such a scenario reads as follows, “You are jogging in the park when a dog starts bounding towards you. As it gets closer you realise it is quite *aggressive*/*playful*” (negative and positive resolution respectively; Holmes and Mathews 2005). Using this methodology it has been demonstrated that repeated exposure to positive imagery scenarios contributes to the acquisition of a positive interpretive bias (Holmes et al. 2006), while repeated exposure to negative imagery scenarios contributes to the development of an interpretive bias favouring negative resolutions of ambiguous material (Holmes and Mathews 2005). Subsequent studies have confirmed that imagery-based approaches to CBM-I are capable of consistently modifying interpretive selectivity (Lau et al. 2011). Furthermore, initial research with clinically depressed populations has highlighted potential applied value of these tasks for altering the problematic patterns of interpretation which characterise depression (Blackwell and Holmes 2010; Lang et al. 2012).

Given these encouraging findings with imagery-based CBM-I, identifying the precise mechanisms that serve to facilitate interpretive change is crucial to establishing the most effective means of ameliorating biased interpretation and emotional vulnerability. However, few studies have sought to systematically investigate the way in which imagery tasks serve to modify interpretive bias. The rationale for achieving change in selective interpretation via imagery-based CBM-I suggests that repeatedly picturing oneself in ambiguous scenarios that are then resolved either positively or negatively will modify patterns of biased interpretation regarding the expected outcome of subsequent events. The result is that when novel ambiguous information is encountered, the individual will apply the same pattern of interpretation represented in the imagery scenarios (e.g. Holmes et al. 2006; Lau et al. 2011). While this ambiguity resolution account of imagery-based CBM-I is entirely plausible, an alternative, and as yet untested possibility, is that the emotional valence of the imagined scenarios could produce such change without the necessary presence of ambiguity.

With imagery-based CBM-I it is assumed that the initial ambiguity of scenarios and their subsequent resolution is necessary for altering biased interpretation. However, research suggests that imagery has the capacity to exert a profound impact on emotion even in the absence of ambiguity (Borkovec et al. 1993; Holmes and Hackmann 2004). Findings suggest that the ability to produce vivid mental representations of emotionally negative or positive events, can influence judgments about the likelihood that these events will occur (Carroll 1978; Sherman et al. 1985). It is possible therefore, that, regardless of its inherent ambiguity, the act of repeatedly producing a vivid positive or negative mental image of a situation could systematically bias interpretation in line with the emotional valence of the imagined events.

Such a possibility has parallels with the theoretical proposition originally proposed by Grey and Mathews (2000) suggesting that repeated exposure to positive or negative scenarios may result in a generic emotional priming effect whereby subsequently encountered ambiguous information is interpreted in a valence-congruent manner. This highlights the possibility that a training task may exert an impact on measures of interpretive bias without actually influencing the learning processes involved in the resolution of ambiguity. Some prior research has sought to examine this possibility in non-imagery CBM-I methodologies. Such studies have compared the acquisition of interpretive bias via ‘passive’ CBM-I training (involving reading complete scenarios) to the acquisition of interpretive bias via ‘active’ CBM-I training (involving the active resolution of scenarios; Hoppitt et al. 2010a, b). These studies have produced mixed results however, with one finding induced interpretive bias effects only in the active condition (Hoppitt et al. 2010a) while the second found no effects of induced interpretive bias for either active or passive training (Hoppitt et al. 2010b).

Thus, with respect to imagery-based CBM-I, if ambiguity is not necessary to modify biased interpretation and the emotional valence of imagined scenarios alone can produce measurable differences in interpretive bias, this would suggest that the effects derived from imagery-based CBM-I may be due to the type of emotion-congruent priming proposed by Grey and Mathews (2000), rather than the systematic modification of a decision mechanism that directs the resolution of emotional ambiguity. No research to date has sought to establish if the emotional valence of imagery scenarios is sufficient to modify patterns of biased interpretation.

The principal purpose of the current study was to examine whether ambiguity in imagery-based CBM-I stimuli is necessary to alter patterns of biased interpretation. Consistent with the rationale outlined above, it is possible that the presence of initial ambiguity is not in fact necessary for modifying interpretive bias and instead, mere exposure to scenarios with emotional content is sufficient. This view suggests that imagining positive scenarios involving playful dogs (as per the example above) and other consistently positive (or negative) situations will produce measurable differences in interpretation bias without any need for initial ambiguity. This we refer to as the ‘Emotional Valence Account’ of imagery-based CBM-I. Alternatively, it is possible that the emotional content of imagined scenarios alone cannot change biased interpretation. This instead suggests that the presence of emotional ambiguity and its final resolution, is critical to modifying patterns of selective interpretation in imagery-based CBM-I. According to this view no amount of imagining playful dogs, sunshine, lollipops, rainbows or other positive (or negative) events will influence biased interpretation unless the description of these events also incorporates initial ambiguity. This we refer to as the ‘Ambiguity Resolution Account’ of imagery-based CBM-I.

To test these alternative accounts of interpretive bias modification, the present study delivered four different imagery scenario conditions. The same imagery scenarios used by Holmes et al. (2006) were adapted into four different versions which varied according to the emotional valence of the scenario and the presence of ambiguity in the scenario. Participants therefore were exposed to either positive or negative imagery scenarios (positive vs. negative scenario conditions respectively), where the ambiguity was either present, such that the valence of the scenario only became apparent in the final word/s (as per Holmes et al. 2006, original task) or, the ambiguity was absent, such that the emotional valence of the scenario was clear from the beginning (ambiguity present vs. ambiguity absent conditions respectively). Table 1 provides two examples of the manner in which the scenarios were altered to either preserve or remove emotional ambiguity.

**Table 1:** Example scenarios demonstrating alternate orders of emotional resolution in ambiguity present and ambiguity absent scenario conditions (alternative emotional resolutions given in italics)

	Ambiguity present		Ambiguity absent	
	Beginning	End	Beginning	End
E.g. 1	You have been to the dentists for a filling to your back molar. You have had a local anaesthetic but after it wears off…	…you find you are in *pain*/*no pain*	You find you are in *pain/no pain…*	…when a local anaesthetic wears off after having been to the dentists for a filling to your back molar
E.g. 2	You are skiing down a slalom slope at high speed. You fall and hear a crack	You realise that you have broken a *bone/ski*	You realise that you have broken your *bone/ski*	…when you fall and hear a crack. You had been skiing down a slalom slope at high speed

To assess changes in biased interpretation, ten emotionally ambiguous test scenarios were dispersed within the latter half of the imagery task. These scenarios remained ambiguous in that their emotional valence was not resolved. Two measures assessed the interpretation imposed on these scenarios. The first was participant’s emotionality ratings of the ambiguous test scenarios (consistent with; Berna et al. 2011). The second was derived from a subsequent recognition memory task in which participants rated the similarity of positively and negatively disambiguated versions of the ambiguous test scenarios (consistent with; Mathews and Mackintosh 2000).

If the emotional valence of the scenario alone is sufficient to modify interpretation (consistent with the Emotional Valence Account), then we would expect participants in both the ambiguity present and ambiguity absent conditions to adopt an interpretive bias in line with their allocated scenario valence condition. Such a pattern of findings would implicate the role of valence-congruent priming in the patterns of acquired interpretive bias commonly observed with imagery-based CBM-I. However, if scenario ambiguity is critical to the acquisition of an interpretive bias in imagery-based CBM-I (consistent with the Ambiguity Resolution Account), then we would instead predict that only participants in the ambiguity present condition would acquire an interpretive bias in line with the valence of the scenarios. This instead would suggest that imagery-based CBM-I exerts its influence on a more underlying decision mechanism that informs the resolution of emotional ambiguity.

## Method

### Participants

To reduce the likelihood that participants had a strong existing positive or negative interpretive bias, participant selection was guided by initial screening of 840 first year undergraduate students on the trait version of the Spielberger State Trait Anxiety Inventory (STAI-T Spielberger et al. 1983). Participants were considered eligible to participate if their scores fell within the middle third of the distribution (STAI-T = 36–44 inclusive). Of those eligible, the first 80 to accept an invitation to participate were included in the study and were randomly assigned to one of the four scenario conditions. The total sample comprised 26 male and 54 female participants with a mean age of 18.93 years (SD = 4.84). One-way analysis of variance confirmed that participants did not significantly differ across the four experimental groups in terms of age, STAI-T score, or STAI-S (all *F*’s < 1), assessed at time of testing. Similarly, Chi square analysis revealed that gender ratios did not differ significantly across the four conditions, χ^2^ (3, 78) = 1.65, *p* = .20. Mean age, STAI-T and gender ratios for each condition are provided in Table 2.

**Table 2:** Participant gender, mean age and STAI-T across experimental groups

Experimental condition	*N*	Gender M/F	Age (in years)	STAI-T	STAI-S
Ambiguity present					
Positive	20	5/15	17.85 (1.50)	46.65 (3.87)	43.95 (5.32)
Negative	20	6/14	19.70 (5.49)	46.05 (4.10)	45.55 (4.71)
Ambiguity absent					
Positive	20	9/11	19.40 (6.62)	45.91 (4.01)	43.40 (5.55)
Negative	20	6/14	18.75 (4.36)	46.10 (3.88)	44.75 (6.19)

Standard deviations given in parentheses

### Materials

#### Emotional Assessment

State and trait anxiety were assessed using the Spielberger State-Trait Anxiety Inventory (STAI; Spielberger et al. 1983). Each of the two subscales consists of 20 items, with higher scores indicating higher levels of state (STAI-S) or trait (STAI-T) anxiety. The STAI has demonstrated validity and reliability across a range of populations (Barnes et al. 2002). Current mood was assessed after the completion of the emotional scenarios using a Visual Analogue Mood Scale. This consisted of three positive mood items (excited, happy, enthusiastic) and three negative mood items (distressed, irritable, and anxious) that were each rated according to how the participant was feeling at that moment from 1 (not at all) to 9 (extremely). Items from these analogue mood scales were summed to yield two composite scores for positive and negative mood. The items: distressed, irritable, and anxious were therefore summed to yield a negative affect score and the three positive mood items: excited, happy, and enthusiastic were summed to create a positive affect score.

#### Imagery Scenarios

Each experimental condition included 100 pre-recorded auditory scenarios (110 including ambiguous test scenarios) derived from those used by Holmes et al. (2006), and adapted for Australian cultural norms. The type of situation depicted in each scenario was identical across the four conditions; however the scenarios differed in terms of (1) emotional valence, and (2) the presence of emotional ambiguity. Across the positive and negative scenario valence conditions, the imagery scenarios differed only in terms of single word/words which rendered the emotional tone of the scenario either positive or negative respectively. Across the two scenario ambiguity conditions, scenarios differed only according to the presence of emotional ambiguity. For the ambiguity present condition scenarios were designed such they were initially ambiguous and this was only resolved in the final word/s. Conversely, in the ambiguity absent condition scenarios were constructed such that the emotional tone of the situation was clear from the beginning (see Table 1 for examples).

To assess the degree to which participants acquired an interpretive bias in a manner consistent with the emotional valence of the scenarios they were exposed to, ten emotionally ambiguous test scenarios were presented, randomly dispersed within the latter half (final 50) of the imagery scenarios. Test scenarios were delivered in this manner to disguise their purpose among the remainder of the resolved training scenarios. The same ten emotionally ambiguous test scenarios were included for all participants. A number of these scenarios were adapted from those previously employed by Mathews and Mackintosh (2000). These scenarios were emotionally ambiguous in that either a positive or a negative resolution was possible on the basis of the information provided, and no resolution of this ambiguity was offered. An example of one such emotionally ambiguous test scenario reads: “You are trying out some new recipes you found and begin preparing a dish to serve your family that night when your partner comes in and makes a comment about the smell.” Participants could therefore interpret the situation as having either a positive outcome (the food smells delicious) or a negative outcome (the food smells horrible). Two separate measures provided an indication of the interpretation imposed on these scenarios. The first was the emotionality ratings completed immediately after hearing the scenario, and the second was derived from a subsequent recognition memory task.

#### Imagery Task

Digital recordings of all 110 scenarios were played aloud in a male voice, each lasting approximately 10–13 s. These were delivered stereophonically via headphones. Participants were instructed to close their eyes while listening to the scenarios and, during the description, to imagine the events depicted as if they were happening to themselves. These instructions were consistent with those used in previous research to foster a first person perspective (Holmes et al. 2006). A 3 s pause followed the presentation of each scenario to allow participants to complete the mental image of the situation depicted. To ensure that the two scenario ambiguity conditions did not differentially influence how vividly the scenarios were imagined, or how emotional the scenarios were, ratings of vividness and emotionality were completed following each scenario presentation. Vividness was rated on a five-point scale from 1, *perfectly clear and as vivid as normal vision*, to 5, *no image at all*. Emotional valence was rated on a nine-point scale from 1, *extremely unpleasant*, to 9, *extremely pleasant*. Upon completion of the ratings participants pressed the space bar to begin the next scenario presentation.

### Filler Task

A 5 min filler task was delivered between completion of the imagery scenarios and the recognition memory test. This comprised a short arithmetic task in which participants were presented with a string of three randomly generated digits. On each trial participants were instructed to respond to the parity of the majority of digits by pressing the left mouse button when two of the three digits were odd, and the right mouse button when two of the three digits were even. Participant’s responses cleared the screen and initiated the next trial. The number of trials completed varied within the constraint that the task ran for 5 min.

#### Recognition Memory Task

The recognition memory test was designed to assess the interpretations imposed on the ten emotionally ambiguous test scenarios and was similar to that used by Mathews and Mackintosh (2000). All participants received the same 20 recognition memory items, 10 of which related to the critical emotionally ambiguous test scenarios and the remainder related to 10 resolved imagery scenarios which were included to disguise the purpose of the task. For each recognition memory item, participants were presented with a cue regarding the content of the scenario (e.g. “Cooking a new recipe”) along with four different statements. These statements included a possible positive disambiguation, a possible negative disambiguation, a positive foil, and a negative foil. Foil items were included to confirm that participants were not simply responding in a valence-congruent manner. Each individual statement was rated according to its similarity in meaning to the original corresponding scenario on a four-point scale from 1, very similar in meaning, to 4, very different in meaning. Example statements for the scenario “Cooking a new recipe” included: “When trying out a new recipe your partner walks into the kitchen and says that the food smells horrible”(negative disambiguation); “When trying out a new recipe your partner walks into the kitchen and says that the food smells delicious” (positive disambiguation); “Your partner walks into the kitchen while you’re trying out a new recipe and helps you out” (positive foil); “Your partner walks into the kitchen while you’re trying out a new recipe and disturbs you” (negative foil). The order of these individual statements was randomised for each participant, as was the order in which each individual recognition memory item was presented.

#### Procedure

After providing informed consent, participants were randomised to one of the four imagery scenario conditions. Questionnaires measures and all experimental tasks were delivered on a PC with a high resolution 15 inch monitor using E-Prime software (Version 2.0, Psychology Software Tools, Pittsburgh, PA, USA). Participants were seated at the computer and wore headphones for the duration of the experiment. All instructions were displayed on screen and participants were encouraged to ask the experimenter for clarification at any stage if required. Participants initially completed demographic questions and the state and trait versions of the STAI. Before beginning the imagery scenario task participants were provided a description of what was meant by using mental imagery. They were then given two non-emotional practice scenarios and were asked to imagine each situation in a first person manner, as if it were happening to themselves. Participants were instructed to close their eyes while listening to the scenarios to help focus on the image. They then answered the subsequent questions regarding how vividly they were able to picture the scenario and how pleasant or unpleasant the situation depicted was. Following delivery of the imagery scenarios, participants completed the filler task, followed by the recognition memory test. For the recognition memory test participants were initially provided with an example item (related to the imagery example used at the beginning of the imagery task) to illustrate the task. Participants were informed that no single statement necessarily reflected a correct answer and that they should independently rate each according to how similar they felt it was to the original scenario. At the conclusion of the study participants were debrief and thanked for their participation.

## Results

Examination of the data revealed that two participants failed to provide responses for all the required tasks and were therefore excluded from the final analysis. For all remaining experimental measures, no outliers were observed three standard deviations above or below the group mean, and therefore no data were excluded on this basis.

Before addressing the key hypotheses under scrutiny, vividness and emotionality ratings for the 100 experimental scenarios (not including the ten ambiguous test scenarios) were compared across the ambiguity present and ambiguity absent conditions to determine if there were any systematic differences in these ratings that could potentially confound the experimental manipulation. Reassuringly, mean vividness ratings did not significantly differ between the ambiguity present and ambiguity absent conditions, *t*(1, 76) = .70, *p* = .487. Similarly, comparison of emotionality ratings across the ambiguity present and ambiguity absent conditions for the positive scenario condition, *t*(1, 37) = 1.63, *p* = .112, and the negative scenario condition, *t*(1, 37) = .96, *p* = .342 did not reveal any significant group differences. This suggests that the ambiguity manipulation did not produce systematic differences in perceptions of either the vividness or emotional intensity of the scenarios.

### Assessing the Influence of Scenario Ambiguity and Scenario Valence on the Acquisition of Interpretive Bias

To address alternative accounts concerning whether the emotional valence of imagery scenarios is sufficient to produce change in interpretive bias (Emotional Valence Account) or, if the resolution of ambiguity is critical in order to achieve such interpretive change (Ambiguity Resolution Account), data derived from the 10 ambiguous test scenarios delivered in the latter half of the imagery task were examined. In the following analyses we consider each of the dependent measures derived from these ambiguous test scenarios in turn. The first analyses examine the similarity ratings from the recognition memory test, while the subsequent analyses focus on the emotionality ratings of the ambiguous test scenarios (completed during the imagery task).

#### Recognition Memory Task

To assess whether participants acquired an interpretive bias in line with their assigned valence condition, mean similarity ratings of the positively disambiguated recognition memory statements and the negatively disambiguated recognition memory statements for the ten ambiguous test scenarios were examined. These were subjected to a 2 × 2 × 2 mixed model ANOVA with scenario valence condition (positive vs. negative) and scenario ambiguity condition (ambiguity present vs. ambiguity absent) as between-subject factors, and recognition memory statement valence (positive vs. negative) as the within subject factor. If the resolution of ambiguity is not necessary to achieve change in biased interpretation, as suggested by the Emotional Valence Account, then we would expect a significant two-way interaction between scenario valence and recognition memory statement valence, not further modified by scenario ambiguity condition. The nature of this two-way interaction would be such that, participants in the positive valence condition would rate positively disambiguated statements as more familiar than negatively disambiguated statements while those in the negative valence condition would rate negatively disambiguated statements as more familiar than the positively disambiguated statements across both ambiguity conditions. Alternatively, the Ambiguity Resolution Account suggests that the presence of ambiguity is critical to modifying interpretive bias and therefore, a three-way interaction will be observed such that the two-way interaction involving scenario valence and recognition memory statement described would only be evident in the ambiguity present condition and not in the ambiguity absent condition.

**Fig. 1:**
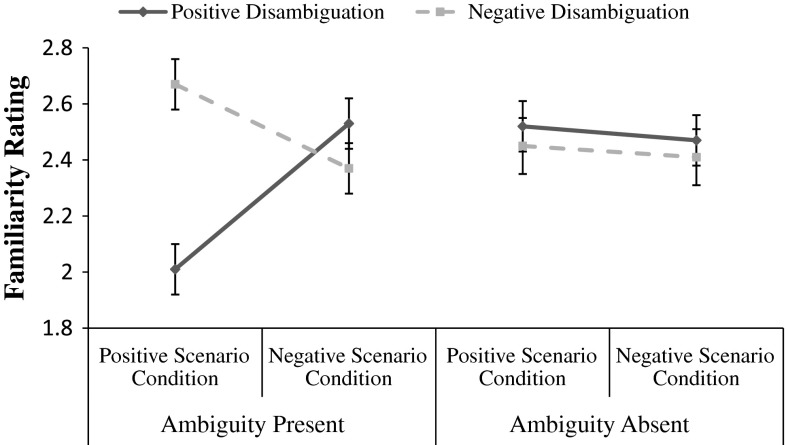
Similarity ratings for positive and negative recognition memory statements across positive and negative scenario conditions. Lower scores represent greater similarity

Consistent with the Ambiguity Resolution Account a significant three-way interaction was indeed observed between scenario valence condition, scenario ambiguity condition, and recognition memory statement valence *F*(1, 74) = 8.79, *p* = .004, η_p_^2^ = .11. Examination of the component two-way interactions for the two ambiguity conditions separately revealed that the three-way interaction comprised a significant two-way interaction in the ambiguity present condition *F*(1, 37) = 22.79, *p* < .001, η_p_^2^ = .38 (see Fig. 1), whereas this interaction was non-significant in the ambiguity absent condition *F*(1,37) < 0.001, *p* = .997. As can be observed in Fig. 1, for those in the ambiguity present condition, participants in the positive scenario condition rated the positively disambiguated statements as more similar in meaning to the original ambiguous test scenario, while those in the negative scenario condition rated negatively disambiguated statements as more similar in meaning to the original ambiguous test scenario. While the direction of the effects was consistent with each of the respective scenario valence conditions, a significant difference between ratings for the positively and negatively disambiguated statements was observed only for the positive scenario condition *t*(1, 19) = 6.27, *p* < .001, and not the negative scenario condition *t*(1, 18) = 1.17, *p* = .256. Thus, while the interaction is entirely consistent with the acquisition of an interpretive bias in line with the valence condition, examination of these component effects suggest that this two way interaction was predominantly carried by a pattern of acquired interpretive bias in the positive condition. These findings also revealed no evidence in the ambiguity absent condition that scenario valence exerted any impact on recognition memory for positively and negatively disambiguated statements.

To confirm that these findings did indeed reflect the acquisition of an interpretive bias in the ambiguity present condition, and not a systematic response bias to positive or negative statements more generally, similarity ratings for the foil statements were included in a subsequent mixed model ANOVA. This comprised a 2 × 2 × 2 × 2 ANOVA with scenario valence condition (positive vs. negative) and scenario ambiguity condition (ambiguity present vs. ambiguity absent) as the between subjects factors, and recognition memory statement valence (positive vs. negative) and recognition memory statement type (disambiguated statement vs. foil statement) as the within subject factors. If similarity ratings represent a systematic response bias rather than a true interpretive bias, we would expect no difference between the pattern of similarity ratings of foil statements and disambiguated statements. This would be demonstrated in a significant three-way interaction reflecting the same pattern of findings described above, that was not modified further by the type of recognition memory statement (disambiguated statement vs. foil statement). However, if it were a true interpretive bias, then, we would expect a significant four-way interaction, comprising a significant three-way interaction for the disambiguated recognition memory statements only, and no three-way interaction involving foil statements. Consistent with the position that present findings represent the genuine acquisition of an interpretive bias, a significant four-way interaction was observed between scenario valence condition, scenario ambiguity condition, type of recognition memory statement, and valence of the recognition memory statement, *F*(1, 74) = 5.09, *p* = .027, η_p_^2^ = .064. Examination of the component three-way interactions revealed the significant interaction for the disambiguated recognition memory statements described above *F*(1, 74) = 8.79, *p* < .01, η_p_^2^ = .11 and no three-way interaction for the foil statements, *F* < 1. Mean and standard deviations for recognition memory ratings across statement type (disambiguated test statements vs. foil), statement valence (positive vs. negative), scenario ambiguity condition (ambiguity present vs. ambiguity absent) and scenario valence condition (positive vs. negative) are provided in Table 3.

**Table 3:** Means and standard deviations for recognition memory ratings across statement type (disambiguated test statements vs. foil), statement valence (positive vs. negative), scenario ambiguity condition (ambiguity present vs. ambiguity absent) and scenario valence condition (positive vs. negative)

Statement type	Statement valence	Ambiguity condition	Valence condition	Mean	SD
Disambiguated test statement	Positive	Ambiguous	Positive	2.03	0.39
			Negative	2.53	0.40
		Unambiguous	Positive	2.52	0.41
			Negative	2.47	0.35
	Negative	Ambiguous	Positive	2.67	0.45
			Negative	2.37	0.33
		Unambiguous	Positive	2.46	0.45
			Negative	2.41	0.42
Foil statement	Positive	Ambiguous	Positive	3.22	0.36
			Negative	3.46	0.30
		Unambiguous	Positive	3.45	0.31
			Negative	3.53	0.40
	Negative	Ambiguous	Positive	3.30	0.38
			Negative	3.37	0.40
		Unambiguous	Positive	3.43	0.49
			Negative	3.47	0.39

#### Emotional Valence Ratings

**Fig. 2:**
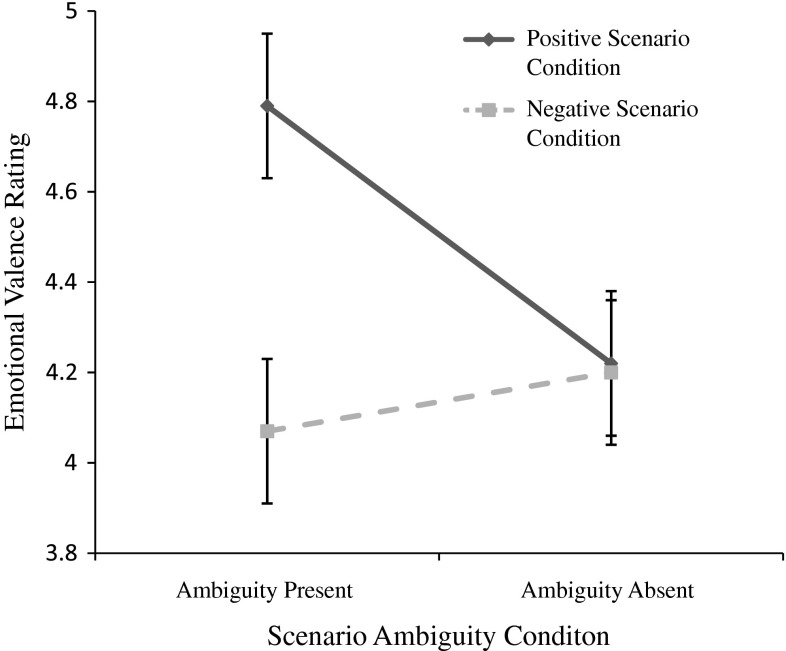
Emotional valence ratings for ambiguous test scenarios across positive and negative, and ambiguity present and ambiguity absent imagery scenario conditions

As an additional test of acquired interpretive bias, emotional valence ratings of the ten ambiguous test scenarios were examined in a 2 × 2 between subjects ANOVA, with scenario valence condition (positive vs. negative) and scenario ambiguity condition (ambiguity present vs. ambiguity absent) as between-subject factors. If these data are consistent with the pattern of findings observed on the recognition memory task then we would expect a two-way interaction between scenario valence condition and scenario ambiguity condition such that different emotional valence ratings would be evident across positive and negative valence conditions in the ambiguity present condition only, and not in the ambiguity absent condition. Results indicated a significant two way interaction between scenario valence condition and scenario ambiguity condition, *F*(1, 74) = 4.61, *p* = .035, η_p_^2^ = .059. As can be observed in Fig. 2, the nature of this interaction was such that, for those in the ambiguity present condition, exposure to the positive scenario condition resulted in significantly more positive ratings of ambiguous test scenarios as compared to those exposed to the negative scenario condition *t*(1, 37) = 2.74, *p* = .009, η_p_^2^ = .17. However, for those in the ambiguity absent condition no significant difference was observed in ratings of emotional valence of ambiguous test scenarios across positive and negative valence conditions *t*(1, 37) = 0.08, *p* = .940. Again, this pattern of findings is entirely consistent with the acquisition of an interpretive bias in the ambiguity present condition, which was not achieved in the ambiguity absent condition in line with the Ambiguity Resolution Account of interpretive bias acquisition.

#### Mood Ratings

Participants completed mood ratings immediately following completion of the imagery task. The composite scores for positive and negative mood were each included as a dependent measure in separate analyses. A 2 × 2 mixed model ANOVA involving the between subjects factors of imagery valence condition (positive vs. negative) and ambiguity (ambiguity present vs. ambiguity absent) did not reveal any significant main effects or interactions for either the positive mood scores (largest *F* = 2.93, smallest *p* = .09) or the negative mood scores (largest *F* = 0.78, smallest *p* = .38). This suggests that neither ambiguity condition produced a systematic group difference in either positive or negative mood across the positive and negative valence conditions.

## Discussion

The aim of the present study was to determine whether the presence of ambiguity in imagery scenarios is critical to modifying biased interpretation, or, if the emotional valence of imagery scenarios alone is sufficient to achieve such bias modification. The observed pattern of findings provides unequivocal support for the position that the presence of initial ambiguity is indeed necessary to alter patterns of selective interpretation. This finding was consistent across both measures of interpretive bias acquisition assessed in the present study. Results of the recognition memory task revealed that those in the ambiguity present condition adopted a pattern of selective interpretation that was entirely consistent with the valence of the scenario training condition to which they were allocated, with those in the negative scenario condition rating negative disambiguations as more similar in meaning, and those in the positive scenario condition rating positive disambiguations as more similar in meaning to the ambiguous test scenarios. By contrast, those in the ambiguity absent condition showed no difference in the pattern of interpretive bias on the recognition memory task. These results were mirrored in the emotional valence ratings of the ambiguous test scenarios where those in the ambiguity present condition produced ratings that were entirely consistent with the scenario valence condition to which they were allocated, while those in the ambiguity absent condition who showed no difference in their pattern of emotional valence ratings across the different scenario valence conditions. This pattern of findings highlights that only those in the ambiguity present condition came to interpret novel ambiguous information in a manner consistent with the emotional valence of the scenarios they had imagined. Furthermore, this strongly suggests that the modification of interpretive bias via imagery-based CBM-I is not achieved by merely imagining positive or negative events of any type. In fact, these findings suggest that simply imagining positive or negative events, in the absence of ambiguity, will have little to no impact on the acquisition of selective interpretation. Instead, the results of the present study underscore the critical nature of ambiguity in modifying biased interpretation.

The support for the Ambiguity Resolution Hypothesis in the present study is consistent with the proposition that it is necessary to alter implicit production rules concerning the resolution of ambiguity in order to modify patterns of biased interpretation. As originally highlighted by Grey and Mathews (2000) interpretive bias can potentially be acquired via different means. One possibility being that repeated processing of information of one valence will increase the accessibility of such information and alter the resolution of subsequently encountered ambiguous information in a valence-congruent manner. Alternatively, it may be necessary to acquire learning of a specific production rule regarding the interpretation of ambiguity by consistently activating alternative meanings which are then resolved in favour of one emotional outcome. Consistent with the latter account, the present findings clearly suggest that the repeated processing of emotional information will only serve to alter the resolution of novel ambiguous material if such information initially activates alternative emotional meanings (i.e. is ambiguous). Inconsistent with the emotional priming perspective however, the present findings suggest that the repeated processing of emotional material that does not activate alternative emotional meanings (i.e. is unambiguous) will not serve to alter the resolution of subsequent ambiguous information. Thus these findings clearly reinforce the position that to alter patterns of selective interpretation, it is necessary for the training material to activate competing alternative meanings with the resolution then consistently favouring one emotional outcome.

While the interaction involving the recognition memory data demonstrated that interpretive bias was only modified in the ambiguity present condition, it was also the case that this effect was carried to a large extent by the positive scenario condition. The findings revealed that there was a significant difference in similarity ratings for positive and negative recognition memory items in the positive scenario condition but not the negative scenario condition. While both the effects were in the expected direction these findings clearly suggest that participants more readily acquired an interpretive bias toward positive resolutions of ambiguity rather than negative resolutions of ambiguity. Other studies have also revealed similar patterns of findings when comparing CBM-I training for positive or negative resolutions. One study found a substantially smaller training effect for interpret negative as compared to the interpret positive CBM-I condition (Salemink et al. 2010) while another found only a trend in the interpret negative condition compared to a significant effect in the interpret positive condition (Salemink and van den Hout 2010). A common feature of both the current study and these prior studies is that they both incorporated non-clinical samples (undergraduate) with mid-range trait anxiety. It is possible, therefore, that amongst such samples participants have relatively low, homogenous interpretive bias, and a greater readiness to acquire a positive bias than to acquire a negative bias. However, with increasing evidence from both CBM-I and other CBM techniques (e.g. Grafton et al. 2012) suggesting that the acquisition of a positive bias may confer greater emotional resilience it is nevertheless encouraging that individuals may show a greater readiness to acquire such a positive bias.

The current pattern of data suggest that participants in the positive condition may have acquired a bias in the targeted direction to a greater degree than those in the negative condition. However, it is worthy to note that any consideration of change in patterns of interpretation in the present study is necessarily speculative as baseline measures of selective interpretation were not included in the current study and as a result, the magnitude of change in bias cannot be compared. Given that some recent findings suggest that individual differences in the readiness to alter patterns of biased cognition may critically underpin changes in emotional vulnerability (Clarke et al. 2012), it would be beneficial for future studies to also include baseline measures of interpretation to allow more precise comparison of the magnitude of change in interpretive bias across different conditions.

The measure of acquired interpretive bias incorporated in the present study represents a common assessment method for scenario-based CBM-I. It is also the case, however, that this recognition memory assessment for novel ambiguous scenarios closely resembles the training task. Thus, the transfer of acquired interpretive bias as demonstrated in the present study represents a ‘close’ transfer effect and conclusions about the generality of such a bias are therefore limited. Similarly, the current study did not seek to examine either the perseveration of the acquired bias, or the impact on emotional reactions to real or contrived stressful experiences. It remains to be seen, therefore, whether the pattern of findings observed in the current study will extend to other assessment tasks and whether changes in emotional vulnerability will be consistent with the changes in biased interpretation. To address the degree of transfer of interpretive bias, future research could incorporate alternative measures of biased interpretation such as homograph priming, or homophone spelling to assess the degree to which imagery-based CBM-I results in such ‘far’ transfer effects. Additionally, lab-based stressor tasks could be incorporated to examine if these CBM-I techniques produce concurrent differences in emotional vulnerability.

While the pattern of effects observed on measures of selective interpretation were entirely consistent with the Ambiguity Resolution Account of interpretive bias acquisition, the present study revealed no evidence of systematic differences in mood across the four experimental conditions. Although the absence of such effects permit confidence that the observed patterns of interpretive bias are unlikely to have been the product of mood state, the lack of mood effects differs from some previous imagery-based CBM-I studies (Holmes et al. 2006, 2009). However, it is apparent that mood does not always change in a manner consistent with acquired interpretive bias following CBM-I with some studies finding no evidence of changes in mood despite the successful modification of interpretive bias (Salemink et al. 2007). The present study included baseline measures of state anxiety using the STAI-S which suggested similarity in these measures across groups. However, no analogue mood scales were completed immediately prior to the CBM-I training. Thus, while change in mood was not a primary focus for the present study, to precisely establish the degree to which the alternative CBM-I conditions produce change in mood, it would be necessary for future studies to include mood measures immediately before and after exposure to the different scenario conditions.

While the ambiguity absent condition found no evidence of interpretive bias acquisition, this very fact could, ironically suggest that this task may have useful applications in future CBM-I research. The potential application of such a task becomes evident when considering the utility of closely matched control conditions in CBM-I research, and cognitive bias modification research more generally. An ideal control condition should incorporate two critical features. Firstly, it should not be capable of modifying the mechanism that is the target of change in the comparative experimental/treatment condition. Secondly, it should be as closely matched in all other respects to the active condition as possible. Research into the potential benefits of cognitive bias modification is generally strengthened by high quality non-treatment control conditions that incorporate many of the same task characteristics as active training/treatment conditions (MacLeod and Mathews 2012). The quality of such control conditions is especially evident when considering the comparatively poorly matched control conditions (such as waitlist) that are commonly employed when examining the effectiveness of other psychotherapeutic treatments (cf. Arch and Craske 2009). Within CBM-I studies participants in active training tend to be exclusively exposed to scenarios that are consistently resolved in a positive manner while those in non-training conditions receive half positive and half negative scenarios (e.g. Hayes et al. 2010). While such a control condition is unlikely to alter the target mechanism (i.e. biased interpretation), the number of positive and negative scenarios encountered in the active as compared to the control condition represents a disparity. Ideally, to be as closely matched as possible, a control condition would involve exposure to the precise same emotional information, in a manner that would not serve to modify interpretive bias. As observed in the current study, the ambiguity absent condition presented the same emotional content but showed no evidence of being able to alter biased interpretation. Further research and replication will obviously be required to confirm that scenarios which remove ambiguity do not modify interpretive bias. It is possible, however, that such scenarios could potentially be utilised as a tightly matched control for an CBM-I task where the same emotional information is presented as an active training condition, but in a manner that does not serve to modify the critical interpretive bias targeted in the training condition. Such a closely matched control could serve to increase confidence that CBM-I tasks achieve change in emotional vulnerability via alteration of specific patterns of selective interpretation and not any more general exposure to emotionally valenced stimuli.

While the current findings strongly suggest that ambiguity is important for altering biased interpretation, the present study did not seek to identify the precise *type* of ambiguity that could most usefully facilitate bias acquisition. Identifying the most appropriate type of ambiguity to incorporate into CBM-I tasks could be critical to enhancing the capacity of therapeutic interventions to ameliorate emotional pathology. There are at least two potentially critical ways in which ambiguous scenarios may be resolved. One possibility is that to most effectively alter interpretive bias, ambiguous scenarios should *equally* implicate positive and negative resolutions based on the initial information provided. This suggests that scenarios should be constructed such that they build to a concluding point where there are two critical competing alternatives that are finally resolved in favour of one meaning. Alternatively, it may be that it is more important for the resolution of a scenario to contradict an initially established expectation. This would instead suggest that the ‘surprise’ value of the ambiguity resolution is important in modifying biased interpretation, as may be implicated by studies that have sought to explicitly target reappraisals of negative events or cognitions (Lang et al. 2009; Woud et al. 2012).

While it is possible that either type of disambiguation could be equally effective in altering patterns of selective interpretation, there is evidence that the resolution of different types of ambiguity may implicate different neurocognitive systems. Research examining the neurocognitive mechanisms involved in the resolution of ambiguity have sought to systematically manipulate the point in a spoken sentence where ambiguous material is resolved (Rodd et al. 2012). Findings also suggest that different cortical regions may be associated with the disambiguation of spoken sentences at different stages (Rodd et al. 2012). That different types of ambiguity register in discrete neurocognitive regions underscores the possibility that alternative types of ambiguity and their resolution may have a different impact the acquisition of interpretive bias. Future research could therefore usefully serve to establish whether interpretive training scenarios which initially equally implicate either positive or negative outcomes prior to their resolution, or if those resolutions which disconfirm an initially established expectation serve to most effectively modify interpretive bias and emotional vulnerability.

While such future research will obviously be required to determine the most effective type of ambiguity that will serve to alter interpretive bias, the results of the present study clearly support the conclusion that the presence of ambiguity in imagery-based CBM-I is critical to modifying patterns of selective interpretation. The absence of group differences in selective interpretation for those exposed to scenarios where emotional ambiguity is absent suggests that the act of merely imagining various positive (or negative) events is unlikely to alter interpretive bias and underscores the importance of preserving such ambiguity in tasks seeking to modify interpretive bias. Thus it seems that no amount of imagining sunshine, lollipops and rainbows will be sufficient to alter biased interpretation unless ambiguity is present.
